# Bioactive natural products from fungicolous Hawaiian isolates: secondary metabolites from a *Phialemoniopsis* sp.

**DOI:** 10.1080/21501203.2014.931309

**Published:** 2014-07-09

**Authors:** Amninder Kaur, Kristina D. Rogers, Dale E. Swenson, Patrick F. Dowd, Donald T. Wicklow, James B. Gloer

**Affiliations:** ^a^ Department of Chemistry, University of Iowa, Iowa City, IA52242, USA; ^b^ Bacterial Foodborne Pathogens & Mycology Research Unit, Agricultural Research Service, National Center for Agricultural Utilization Research, USDA, Peoria, IL61604, USA

**Keywords:** *Phialemonium*, *Phialemoniopsis curvata*, oxirapentyn, gabusectin, destruxin

## Abstract

Chemical investigations of two fungal isolates initially identified as members of the genus *Phialemonium* are described. Both isolates were obtained as colonists of other fungi collected on the island of Hawaii and were later assigned as *P. curvatum*. However, *P. curvatum* has recently been reclassified as a member of a new genus (*Phialemoniopsis*) and renamed as *Phialemoniopsis curvata*. Studies of solid–substrate fermentation cultures of one of these isolates afforded an oxirapentyn analogue and destruxin A_4_ as major components, while analysis of the second strain led to the isolation of several simple aromatic metabolites and a compound of mixed biogenetic origin called gabusectin that had previously been reported only in a patent. Structures were assigned mainly by detailed nuclear magnetic resonance and mass spectrometry analysis, and those of two of the major components were confirmed by X-ray crystallography. This report constitutes the first description of secondary metabolites from a member of the genus *Phialemoniopsis*.

## Introduction

During our ongoing investigations of fungicolous and mycoparasitic fungi as sources of biologically active natural products (Neff et al. 2012), isolates initially identified as members of two different strains of the genus *Phialemonium* were encountered as colonists of wood-decay fungi collected on the island of Hawaii. The genus *Phialemonium* has been considered to be intermediate between *Acremonium* and *Phialophora* and includes fungi that possess morphological features characteristic of both of these genera. Three species of this genus were originally described: *P. curvatum* Gams & McGinnis (conidia allantoid in shape), *P. obovatum* Gams & McGinnis (conidia obovate or ellipsoidal), and *P. dimorphosporum* Gams & Cooke (conidia both allantoidal and obovate) (Gams & Mcginnis 1983), although at least six were later recognized. In recent years, *P. dimorphosporum* has been considered synonymous with *P. curvatum* (Guarro et al. 1999; Perdomo et al. 2011). *Phialemonium* spp., including *P. curvatum*, are known opportunistic human pathogens (Proia et al. 2004; Rivero et al. 2009). *P. curvatum* has also been reported to be antagonistic toward the economically important blue-stain fungus *Ophiostoma crassivaginatum* (Hiratsuka & Chakravarty 1999) and has been suggested as a possible biocontrol agent against blue-stain fungi. Very recently, a report appeared reclassifying *P. curvatum* as a member of a new genus and assigning it the name *Phialemoniopsis curvata* (Perdomo et al. 2013). Neither *Phialemonium* nor its new segregate *Phialemoniopsis* appear to have been explored from a chemical standpoint. The results presented here constitute the first report of secondary metabolites from any member of these taxa.

## Materials and methods

### General experimental procedures

Optical rotations were measured with a Rudolph automatic polarimeter, model APIII. ^1^H and ^13^C nuclear magnetic resonance (NMR) spectra were recorded using Bruker AVANCE-400, AVANCE-500, or AVANCE-600 spectrometers. Chemical shift values were referenced to residual solvent signals for CDCl_3_ (*δ*_H_/*δ*_C_, 7.24/77.2). HMQC, HMBC, and NOESY data were recorded using the Bruker AVANCE-600 instrument. ^13^C NMR multiplicities were not directly measured, but all assignments were fully consistent with HMQC data. HRESITOFMS data were obtained using a Waters Q-ToF Premier mass spectrometer. High-performance liquid chromatography (HPLC) separations were carried out using a Beckman System Gold instrument with a model 166 variable wavelength UV detector connected to a 128 solvent module. Antiinsectan and antifungal bioassays were conducted using previously published protocols (Wicklow et al. 1998; Dowd et al. 2007).

### Fungal isolation and identification

A fungicolous isolate (MYC-2005) was obtained by DTW from the black stromata of a pyrenomycete found on a dead hardwood branch in a coastal mesic Casuarina forest, Mackenzie State Park, Puna District, Hawaii, using general procedures that have been previously described (Höller et al. 2002). A culture of the isolate was deposited at the ARS, USDA culture collection at the NCAUR under accession number NRRL 46124. A subculture of this isolate was later identified as *Phialemonium curvatum* by researchers at the Centraalbureau voor Schimmelcultures (CBS) and deposited in their collection as CBS 121222. After both cultures had been deposited as *P. curvatum*, a recent report (Perdomo et al. 2013) appeared reassigning this species to a new genus (*Phialemoniopsis*) and renaming the species as *Phialemoniopsis curvata*. However, subsequent partial sequence analysis of the internal transcribed spacer (ITS) region and D1 and D2 domains of the nuclear large subunit (28S) rDNA gene using ITS5 and NL4 as polymerase chain reaction and sequencing primers and a nucleotide-to-nucleotide BLAST query of the GenBank database did not afford confirmatory results, leaving some ambiguity about the taxonomic assignment. Sequence information was deposited in GenBank with the accession number GU219470. The BLAST search failed to retrieve a definitive match, and the sequence with the highest similarity score was an unidentified member of the family Bionectriaceae (GenBank accession FJ821507; 98% Ident score).

A second fungal isolate (MYC-1906) similarly collected from an old moldy polypore on a dead hardwood branch in a lowland wet forest, Lava Tree State Park, Hawaii was initially identified as *Phialemonium dimorphosporum.* A subculture of this isolate was deposited at the ARS, USDA culture collection at the NCAUR under accession number NRRL 44611. Partial sequence analysis was carried out using protocols analogous to those noted earlier, and the resulting sequence information was deposited in the GenBank database with the accession number HM060271. In this instance, a BLAST search was much more consistent with the initial micromorphology-based taxonomic assignment. As noted earlier, *P. dimorphosporum* has been considered synonymous with *P. curvatum*, and in view of the recent taxonomic reassignment of *P. curvatum* cited earlier, we now apply the name *Phialemoniopsis curvata* to this strain.

### Fermentation, extraction, and chromatography of NRRL 46124

The tentatively identified isolate of *P. curvata* (MYC-2005 = NRRL 46124; GenBank Accession number GU219470) was grown on 100 g (2 × 50 g) of rice for 30 days at 25°C, and the resulting fermentation was extracted with ethyl acetate (EtOAc). Upon filtration and evaporation, the resulting crude extract (1.4 g) was partitioned between hexanes and CH_3_CN. The CH_3_CN fraction (787 mg) was fractionated on a silica gel column using a hexanes/CH_2_Cl_2_/MeOH solvent system. The column was eluted with 50-mL portions of hexanes-CH_2_Cl_2_ (100:0, 50:50, 0:100) and CH_2_Cl_2_-MeOH (2 × 99:1, 6 × 97:3, 2 × 95:5, 90:10, 75:25 v/v) to afford 15 fractions. Fractions 2–10 eluted with 50% hexanes-CH_2_Cl_2_ through 3% MeOH-CH_2_Cl_2_ and afforded oxirapentyn B (**1**; 130 mg). Fraction 11 was eluted with 3% MeOH-CH_2_Cl_2_ and was further separated using a silica column using a hexanes/EtOAc solvent system. The column was eluted with 100-mL portions of hexanes-EtOAc (100:0, 90:10, 80:20, 70:30, 60:40, 50:50, 40:60, 30:70, 20:80, 10:90, 0:100) and 100 mL MeOH to afford 12 fractions. Fractions 5–6 consisted of an additional sample of **1** (115 mg) and fractions 8–11 contained the known compound destruxin A_4_ (**3**; 65 mg). Oxirapentyn B (**1**) was obtained as a white solid; HRESIMS obsd. *m/z* (M + Na)^+^ 357.1331, calcd. for C_18_H_22_O_6_Na, 357.1314. ^1^H, ^13^C, and HMBC NMR data were consistent with literature values (Yurchenko et al. 2013).

### Oxidation of oxirapentyn B (1)

In an acetone/dry ice bath (−78°C), CH_2_Cl_2_ (75 μL) and oxalyl chloride (6 μL) were added to a 0.5-dram vial. DMSO (5.1 μL) and CH_2_Cl_2_ (15 μL) were added to the oxalyl chloride solution. The resulting solution was stirred for 2 min. Oxirapentyn B (5 mg) in CH_2_Cl_2_ (15 μL) was added within 5 min of the previous step. The solution was then stirred for an additional 30 min. Freshly distilled triethylamine (15 μL) was added and the mixture was stirred for 5 min. The solution was then warmed to room temperature. Water (150 μL) was added, and the aqueous layer was extracted with an additional 150 μL CH_2_Cl_2_. The organic layers were combined and the mixture was purified by reversed-phase HPLC (20% CH_3_CN/H_2_O isocratic for 10 min and 20–100% CH_3_CN over 2 min) with UV detection at 240 nm to afford 3.6 mg of oxirapentyn A (**2**), as verified by comparison of NMR data with literature values (Takahashi et al. 1983).

### Preparation of *p*-bromobenzoate derivative of oxirapentyn B

*p*-Bromobenzoyl chloride (6.1 mg; 0.028 mmol), oxirapentyn B (**1**; 6.6 mg; 0.02 mmol), 4-N,N-dimethylaminopyridine (6.2 mg; 0.051 mmol), and anhydrous CH_2_Cl_2_ (4 mL) were combined in a 20-mL scintillation vial. The resulting solution was stirred at room temperature for 19 h. Saturated aqueous NaHCO_3_ (1 mL) and H_2_O (2 mL) were then added, and the resulting mixture was extracted three times with CH_2_Cl_2_ (total 6 mL). The combined organic phase was evaporated under airflow to obtain a crude product mixture. This mixture was purified by reversed-phase HPLC (50–100% CH_3_CN/H_2_O over 20 min and 100% CH_3_CN over 10 min) with UV detection at 254 nm to afford the *p*-bromobenzoate derivative of **1** (2.2 mg). ^1^H NMR (400 MHz, CDCl_3_) *δ*_H_ 1.27 (s, H_3_-16), 1.43 (s, H_3_-15), 1.43 (dd, *J* = 3.0, 15, H_2_-9) 1.63 (s, H_3_-14), 1.76 (s, H_3_-18), 2.50 (dd, *J* = 3.0, 15, H_2_-9), 3.00 (br s, H-6), 3.45 (br s, H-3), 4.50 (br s, H-4), 4.90 (t, *J* = 3.0, H-8), 5.09 (dq, *J* = 1.5, 1.5, H-13), 5.13 (dq, *J* = 1.5, 1.0, H-13), 5.93 (br s, H-1), 7.61 (d, *J* = 8.5, H-4ʹ and H-6ʹ), 8.02 (d, *J* = 8.5, H-3ʹ and H-7ʹ). Position numbers used in this listing were chosen to match those of the original report of oxirapentyn A (Takahashi et al. 1983).

### X-ray crystallographic data for the *p*-bromobenzoate derivative of oxirapentyn B

A colorless thin plate (0.20 × 0.11 × 0.11 mm), obtained from 2:2:1 CH_3_CN/CHCl_3_/H_2_O, was mounted with paratone oil on the tip of a MiTeGen polymeric mount, and placed on the diffractometer with the long crystal dimension (unit cell body diagonal) approximately parallel to the diffractometer phi axis. Data were collected with a Nonius KappaCCD diffractometer (Mo K-alpha radiation, graphite monochromator) at 210(2) K (cold N_2_ gas stream) using standard CCD techniques yielding 15872 data. Lorentz and polarization corrections were applied. A correction for absorption using the multi-scan technique was also applied (*T*_max_ = 0.983, *T*_min_ = 0.714). Equivalent data were averaged yielding 2536 unique data (*R*-int = 0.078, 1892 *F* > 4σ(*F*), Friedel pairs not averaged). Based on a preliminary examination of the crystal, the space group P2_1_ was assigned (no exceptions to the systematic absence: 0*k*0, *k* = odd were noted). The computer programs from the HKL package were used for data reduction. The preliminary model of the structure was obtained using XS, a direct methods program. Least-squares refining of the model versus the data was performed with the XL computer program. Illustrations were made with the XP program and tables were made with the XCIF program. All of these programs are included in the SHELXTL v6.1 package. Thermal ellipsoids shown in the illustration are at the 50% level. All non-hydrogen atoms were refined with anisotropic thermal parameters. All hydrogen atoms were included with the riding model using the XL program default values. Several low-angle reflections were omitted from refinement due to beam-stop shadowing effects. Data above 21° theta were excluded from the final cycles of refinement as <5% of reflections were above background levels. The bromobenzoyl moiety showed high thermal motion and was modeled with two sites of half occupancy having the benzene rings as rigid groups. Half-atoms (C2ʹ, C2″; C3ʹ, C3″; etc.) were constrained to have the same anisotropic displacement parameters, the groups with C1ʹ were restrained to be flat, C-Br bonds were restrained to be the same, and C1ʹ–C2(‘,”) bonds were also restrained to be the same. No further restraints or constraints were imposed on the refinement model. Crystallographic data for this compound have been deposited with the Cambridge Crystallographic Data Centre (CCDC), with deposition number CCDC 987892. Copies of the data can be obtained, free of charge, on application to the Director, CCDC, 12 Union Road, Cambridge CB2 1EZ, UK (fax: +44-(0) 1223-336033 or email: deposit@ccdc.cam.ak.uk).

### Fermentation, extraction, and chromatography of *P. curvata* NRRL 44611

The second isolate (*P. curvata* MYC-1906 = NRRL 44611; GenBank Accession number HM060271) was grown on 100 g of rice for 30 days at 25°C, and the resulting fermentation cultures were extracted with EtOAc to yield 2.5 g of crude extract. This crude extract was initially partitioned between hexanes and CH_3_CN to afford 240 mg of a CH_3_CN–soluble fraction that was subjected to silica gel column chromatography employing a step gradient of hexanes, EtOAc, and MeOH (100% hexanes; 10, 20, 30, 50, 70 EtOAc in hexanes; 100% EtOAc; 5, 10, 25, 50, and 75% MeOH in EtOAc; 100% MeOH) to afford 15 fractions, which were combined to give 8 fractions based on TLC results. Fraction 3 (23 mg) from the silica column was separated by RP-HPLC (gradient elution using CH_3_CN in H_2_O: 20% for 2 min, 20–80% for 14 min, and 80–100% for 2 min, *λ* = 278 nm) to afford *p*-hydroxyphenethyl alcohol (*t*_R_ = 12.0 min), 3-methoxy-4-hydroxycinnamic acid (*t*_R_ = 13.0 min), indole-3-acetic acid (*t*_R_ = 15.5 min), and gabusectin (**4**; 11 mg). Compound **4** did not exhibit strong absorption in the UV spectral region, so it did not correspond to a major peak in the HPLC chromatogram, but instead was the fraction that was left after small aromatics that showed strong absorptions were separated during the HPLC run. In addition, *p*-hydroxybenzyl alcohol (2.3 mg) precipitated out of fraction 4 (24 mg). Hydroxycinnamic acid, indole-3-acetic acid, *p*-hydroxybenzyl alcohol, and *p*-hydroxyphenethyl alcohol were identified by straightforward analysis of their ^1^H and ^13^C NMR data. Gabusectin (**4**) was obtained as colorless crystals from 1:1 MeOH/H2O; ^1^H, ^13^C, and HMBC NMR data (Table 1) are consistent with partial data presented in the patent report (Schiell et al. 2003): [*α*]^22^_D_  − 87 (c 0.3, MeOH); UV maxima (MeOH) 290, 231 nm; ECD (MeOH) 287 (+2.5), 260 (−4.9), HRESIMS obsd. *m/z* 444.2749 [M + H]+ (calcd. for C_26_H_38_NO_5_, 444.2750).

**Table 1.** ^1^H and ^13^C NMR data for gabusectin (**4**) in CDCl_3_^a^.

Position	*δ*_C_	*δ*_H_, mult (*J* in Hz)	HMBC
1	49.4		
1-Me	21.0	1.23, s	1, 2, 14
		1.22, s	
2	46.1, 46.0	3.34, br d (10)	3, 4, 12
3	133.20, 133.17		
3-Me	23.5	1.66, s	2, 3, 4
4	130.3	5.01, br s	2, 3-Me, 5, 6, 10
5	37.9		
5-Me	32.1	0.67, s	4, 5, 6
6	52.0	1.34, m	7 (wk), 10 (wk)
		0.87, m	4, 5, 5-Me, 7, 7-Me, 8
7	29.7	1.26, m	
7-Me	22.6, 22.5	0.77, d (6.8)	6, 7, 8
8	35.3	1.61, m	
		0.87, m	5- Me, 7
9	25.8	1.80, m	7 (wk), 8 (wk), 10 (wk)
		1.29, m	10 (wk)
10	42.5, 42.4	2.64, dd (12, 4.2)	1, 2, 4, 5, 5-Me, 9, 14
		2.54, dd (12, 4.2)	1, 2, 4, 5, 9, 14
11	132.7	5.29, m	2 (wk), 12, 13
12	129.0	5.40, m	2, 11, 13
13	18.0	1.67, s	12, 13
14	203.4, 203.3		
15	99.2, 99.1		
16	177.5, 177.4		
N-Me	27.4	2.97, s	16, 18
18	64.7	3.68, m	19, 20, 21
19	190.5, 190.4		
20	23.9, 23.8	2.28, m	18, 22
		2.04, m	18, 19, 21, 22 (wk)
21	28.0	2.28, m	18, 20, 22
22	177.9, 177.7		

Notes: ^a^Data were collected at 600 MHz (^1^H, HSQC, and HMBC) and 125 MHz (^13^C); wk = weak correlation. Signals listed with dual entries were those that showed discernible doubling due to the presence of a tautomeric mixture. Mult refers to multiplicity.

### X-ray crystallographic analysis of gabusectin (4)

A colorless plate (0.27 × 0.25 × 0.04 mm) was isolated from the sample and mounted with grease on the tip of a nitrile polymer mount wrapped around a stainless steel pin epoxied to a brass pin and placed on the diffractometer with the long crystal dimension (unit cell ab-face diagonal) approximately parallel to the diffractometer phi axis. Data for **4** were collected with a Nonius Kappa CCD diffractometer (Cu K-alpha radiation, graphite monochromator) at 150(2) K (cold N_2_ gas stream) using standard CCD techniques, yielding 18806 data. Lorentz and polarization corrections were applied. A correction for absorption using the multi-scan technique was applied (*T*_max_ = 0.975, *T*_min_ = 0.844). Equivalent data were averaged, yielding 3177 unique data (*R*-int = 0.051, 1892 *F* > 4σ(*F*); Friedel pairs not averaged). Based on a preliminary examination of the crystal, the space group P4_3_2_1_2 was assigned (no exceptions to systematic absences: h00,h not divisible by 4, 0k0,k = odd were noted). The computer programs from the HKL package were used for data reduction. The preliminary model of the structure was obtained using XS, a direct methods program. Least-squares refining of the model vs. the data was performed with the XL computer program. Illustrations were made with the XP program and tables were made with the XCIF program. All are in the SHELXTL v6.1 package. Thermal ellipsoids shown in the illustrations are at the 50% level. All non-H atoms were refined with anisotropic displacement parameters, and H atoms were included with the riding model using the XL program default values. No further restraints or constraints were imposed on the refinement model. The X-ray data for **4** have been deposited at the Cambridge Crystallographic Data Centre with deposition number CCDC 987893.

## Results and discussion

### Metabolites from NRRL 46124

One of the isolates encountered (MYC-2005 = NRRL 46124) was obtained from the black stromata of a pyrenomycete found on a dead hardwood branch in a forest in Mackenzie State Park, Hawaii and was initially identified as *Phialemonium curvatum* (=*Phialemoniopsis curvata*). The EtOAc extract of rice fermentation cultures of this isolate exhibited antifungal activity against *Fusarium verticillioides* and antiinsectan activity against fall army worm (*Spodoptera frugiperda*) in our assays and was selected for chemical studies. The extract was defatted by partitioning between MeCN and hexanes, and the MeCN-soluble fraction was subjected to silica gel column chromatography using hexanes/EtOAc/MeOH to afford a compound eventually identified as **1** as the most abundant component of the defatted extract. Compound **1** is a reduced (dihydro) analogue of a compound originally called oxirapentyn (**2**), but which was renamed oxirapentyn A as part of a 2013 report describing three other analogues from a marine isolate of *Isaria felina* (Yurchenko et al. 2013). One of these, called oxirapentyn B, afforded spectroscopic data matching those of **1**.

**Figure:**
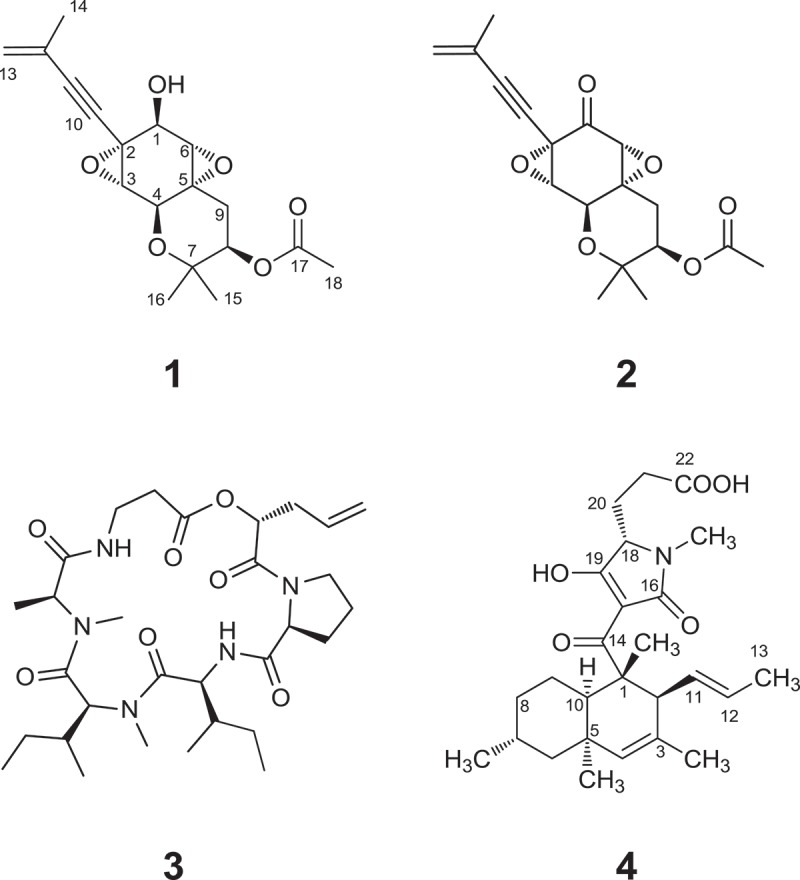

The known cyclohexadepsipeptide destruxin A_4_ (**3**) was also encountered as a major constituent, although in lesser quantities than **1**. The ^1^H NMR spectrum of compound **3** was characteristic of the destruxin class of compounds, and the structure was identified by comparison of the ^1^H NMR data to literature values that had previously been reported by our group in a collaborative study of an entomopathogenic isolate of *Aschersonia* sp. (Krasnoff et al. 1996). Destruxins are composed of five amino acid residues and one α-hydroxy acid unit and are well known for their potent insecticidal and phytotoxic effects (Pedras et al. 2002). It was ultimately determined that compound **3** accounted for the antiinsectan activity observed in our assays.

The molecular formula of **1** was determined to be C_18_H_22_O_6_ (eight unsaturations) on the basis of HRESIMS data. The ^1^H NMR spectrum indicated the presence of a terminal olefin, four oxygenated methines, one vinyl methyl, one acetyl methyl, and two additional methyl groups. The ^13^C NMR data showed signals for 18 carbons, including an ester or acid, two olefinic carbons, eight oxygenated sp^3^ carbons, four methyl carbons, two oxygenated sp^3^ or alkyne carbons, and only one other nonoxygenated sp^3^ carbon.

On the basis of these NMR and MS data, a database search led to the tentative identification of **1** as a reduced (dihydro) analogue of the known compound oxirapentyn (= oxirapentyn A; **2**) (Takahashi et al. 1983), differing only in the absence of a ^13^C NMR signal near *δ* 200 corresponding to the ketone moiety of **2**, along with the presence of an exchangeable OH proton signal and an additional oxymethine signal in the ^1^H NMR spectrum of **1**. The structure was confirmed by detailed analysis of HMQC and HMBC NMR data. After this process, a report appeared describing this compound and assigning it the name oxirapentyn B (Yurchenko et al. 2013). Because the NMR data match closely with those of the literature report, they are not presented here. The absolute configuration of **1** was proposed in that report as shown on the basis of a Mosher ester analysis. However, the relative configuration at the C-6 position was difficult to verify by standard NOESY analysis. Therefore, efforts that had been independently undertaken, which do not duplicate those in the literature and which ultimately verified this assignment, are briefly summarized here.

Oxidation of **1** was carried out under Swern conditions to afford the known compound oxirapentyn A (**2**), as judged by comparison of the NMR data for the product with literature values for **2**. The specific rotation of the oxidation product ([*α*]_D_ = −68) was somewhat different from that reported in the literature for **2** ([*α*]_D_ = −112), whose absolute configuration had been proposed on the basis of CD data. Although the magnitudes did not match, the identity of the NMR data for the product with those of the literature compound, together with the matching sign of the specific rotation, and the small scale on which the reaction was carried out supported assignment of the absolute configuration of **1** at stereocenters shared with **2**.

**Figure 1.**
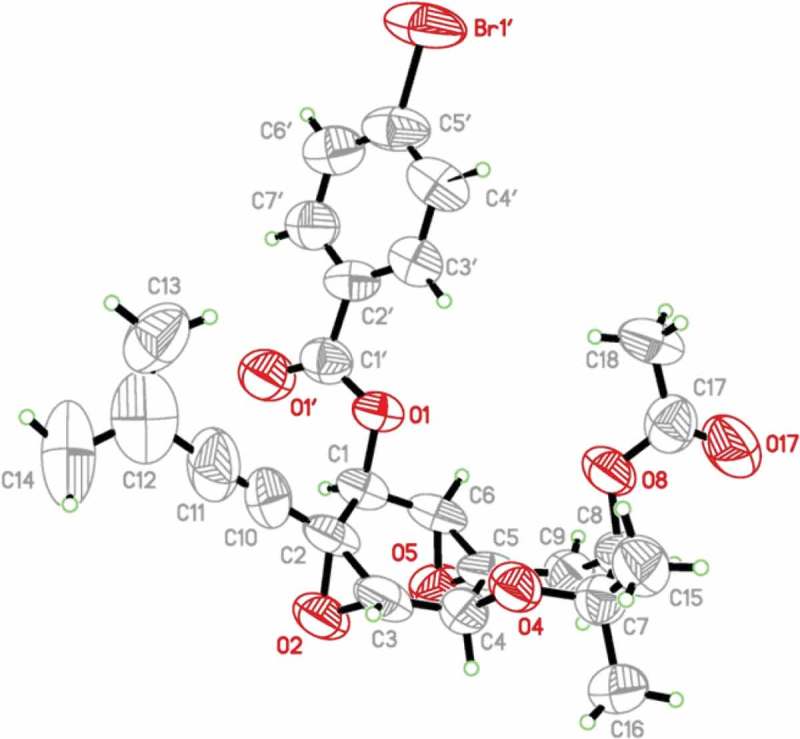
X-ray crystal structure (ORTEP image) of the *p*-bromobenzoate derivative of oxirapentyn B.

While the above mentioned data collectively and consistently suggested the 1*S* configuration for **1**, the perceived stereochemical ambiguity led to efforts to prepare and crystallize the *p*-bromobenzoate derivative of **1** in an effort to independently verify the absolute configuration. Ultimately, suitable crystals were obtained from 2:2:1 CH_3_CN/CHCl_3_/H_2_O. X-ray diffraction analysis enabled independent confirmation of the absolute configuration as shown, including the *S*-configuration at C-1. An ORTEP plot of the crystal structure is shown in Figure 1.

Oxirapentyns (e.g., **1** and **2**) are members of a relatively rare class of highly oxygenated, prenylated cyclohexanoid fungal natural products that include asperpentyn and 7-desoxypanepoxydol. Compound **1** is presumably constructed of a benzenoid aromatic precursor with incorporation of two modified isoprene units at positions C-2 and C-5 of the resulting modified cyclohexane ring. Oxirapentyn A (**2**) was originally reported as a metabolite of the fungus *Beauveria felina*, while oxirapentyn B and two other analogues were later reported from a marine isolate of *Isaria felina* (synonymous with *Beauveria felina*). *Beauveria* is not closely related phylogenetically to *Phialemonium*, but destruxin analogues have also been previously reported from a marine-derived isolate of *B. feline* (Lira et al. 2006). Oxirapentyn A was reportedly active against the Gram-positive bacteria *Staphylococcus aureus* (FDA 209P JC-1), *Bacillus subtilis* (PCI 219), and *Micrococcus luteus* (PCI1001) with MIC values of 25 μg/mL and inactive against *E. coli* (NIHJ JC-2). However, in the present study, oxirapentyn B (**1**) did not show activity in standard disk diffusion assays when tested against *F. verticillioides, Candida albicans* (ATCC 14053), *S. aureus* (ATCC 29213), *B. subtilis* (ATCC 6633), or *E. coli* (ATCC 25922) at 200 μg/disk. As previously mentioned, the antiinsectan activity observed for the original crude extract was due to the known antifungal and phytotoxic compound destruxin A_4_ (**3**) present in the extract, as a dietary concentration of 420 ppm resulted in 67% mortality of *S. frugiperda* at 2 days and 100% mortality after 5 days.

### Metabolites from *P. curvata* NRRL 44611

A second fungal culture collected from an old moldy polypore on a dead hardwood branch in a lowland wet forest, Lava Trees State Park, Hawaii was later identified as *P. curvata* and assigned the culture number MYC-1906 (= NRRL 44611; GenBank Accession number HM060271). Given the results described earlier for an isolate of *P. curvata* obtained from a separate Hawaiian site, a rice fermentation extract prepared from a subculture of this isolate was subjected to chemical investigation, resulting in the isolation of four simple, low molecular weight aromatic compounds: hydroxycinnamic acid (ferulic acid), indole-3-acetic acid, *p*-hydroxybenzyl alcohol, and *p*-hydroxyphenethyl alcohol, along with a compound that has appeared to date only in the patent literature, gabusectin (**4**), that was identified only after analysis of a complete set of 2D NMR data.

Gabusectin (**4**) was assigned the molecular formula C_26_H_37_NO_5_ (nine unsaturations) on the basis of HRESIMS data. While these data clearly indicated the presence of 26 carbon atoms, the ^13^C NMR spectrum (Table 1) showed more than the expected number of resonances due to duplication of some of the signals. Moreover, the ^1^H NMR spectrum (Table 1) showed unusually broad peaks at room temperature. These issues led to some difficulties in the structure elucidation process.

**Figure 2.**
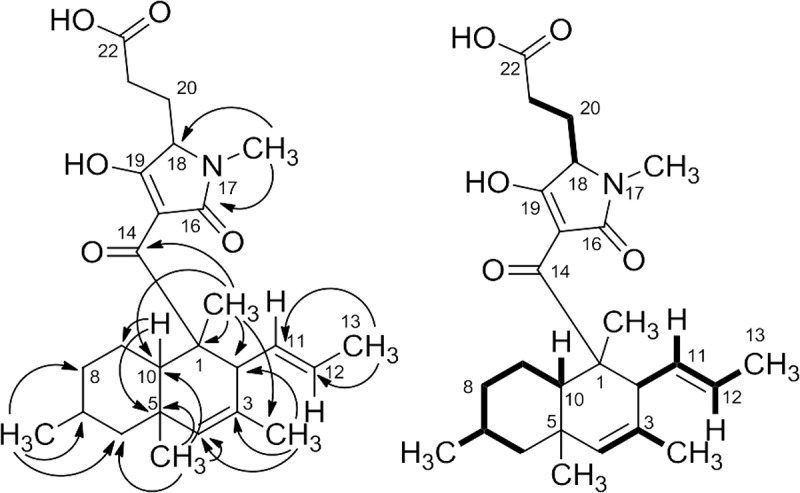
Key HMBC (→) and COSY (▬) correlations for gabusectin (**4**).

Analysis of COSY, HSQC, and HMBC data enabled the identification of a polyketide-derived, modified decalin ring system and assignment of the NMR signals (Table 1). HMBC correlations from the methyl groups alone (Figure 2) were particularly useful in establishing almost the entire structure of this subunit. Connections between CH_2_–8, CH_2_–9, and CH–10 completed this portion of the structure, but could only be established after analysis of COSY data due to broadness, overlap, and signal doubling in the ^1^H and ^13^C NMR spectra. HMBC correlations observed from CH_3_–17 to C–16 and from CH_3_–1 to C–14, as well as the chemical shifts of C–14 (*δ*_C_ 203.4/203.3), C–15 (*δ*_C_ 99.2/99.1), C–16 (*δ*_C_ 177.5/177.4), and C–19 (*δ*_C_ 190.5/190.4) indicated the presence of a tetramic acid unit. These doubled NMR signals for the tetramic acid unit of **4** were recognized as being very similar to those of other related compounds such as equisetin (Vesonder et al. 1979), phomasetin (Singh et al. 1998), and trichosetin (Marfori et al. 2002).

Facile tautomerization associated with the presence of this unit is known to cause signal doubling and broadening in this class of compounds and explained the peak broadening/doubling observed in the ^1^H and ^13^C NMR spectra of **4** (which is shown in only one of its possible tautomeric forms). The CH_2_CH_2_OH side chain of the tetramic acid unit was also identified by analysis of COSY data and suggests that the corresponding portion of the molecule was derived from a glutamic acid unit. Other members of this class, such as those noted earlier, are derived from other amino acid units. Once the gross structure was completed, a literature search identified this compound as a patented metabolite from an unidentified microbial source, which had been given the name gabusectin (**4**) (Schiell et al. 2003). The NMR data for our sample matched closely with data presented in the patent, although signal doubling was not noted or discussed in the report.

**Figure 3.**
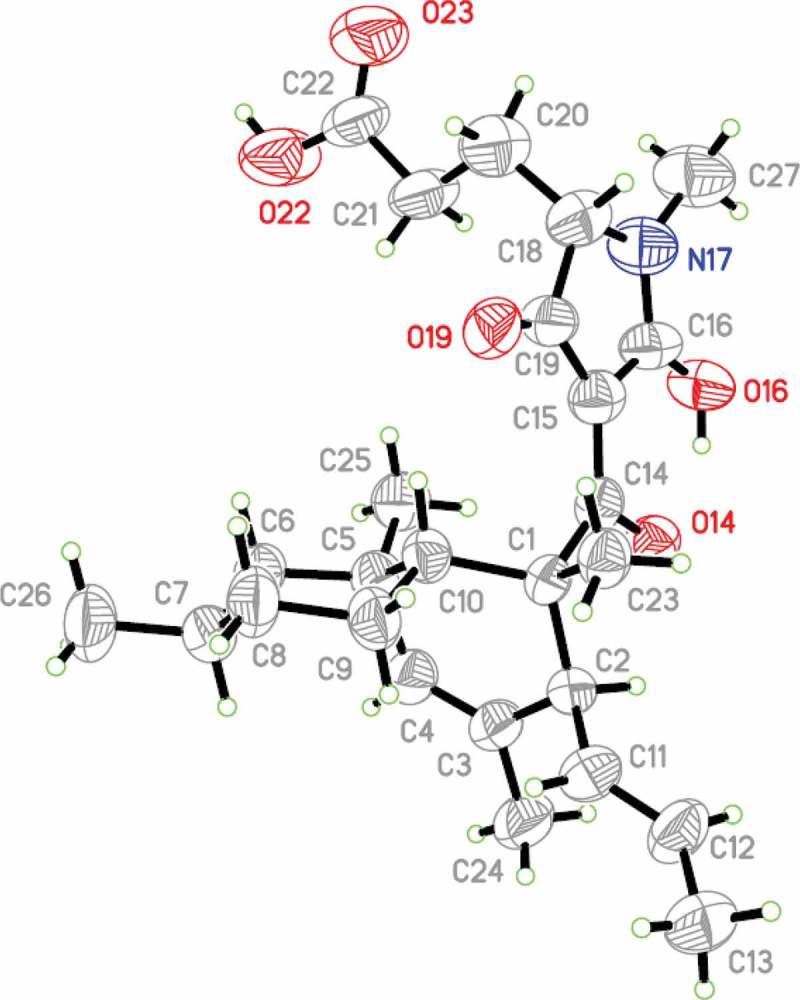
X-ray crystal structure (ORTEP image) of gabusectin (**4**).

Literature reports for these types of compounds indicate that the NMR spectra tend to resolve and sharpen when measurements are made at lower temperature (Singh et al. 1998). However, ^1^H NMR spectra acquired at −20°C and −30°C for our sample of **4** did not show any noticeable improvement. The relative configuration for the substituted decalin unit of **4** was assigned in the patent, but our attempts to verify with confidence that our sample was a match on the basis of NOESY data proved to be unsuccessful due to overlap of key ^1^H NMR signals. The configuration for the glutamic acid-derived unit was not assigned in the patent, and our NMR data also did not enable an assignment. Fortunately, a suitable crystal was obtained from the tautomeric mixture of **4** using 1:1 MeOH/H_2_O as the solvent system. X-ray diffraction analysis enabled assignment of the overall relative configuration (Figure 3), which is consistent with the partial configuration assignment described in the patent and incorporates a *cis*-ring fusion of the decalin moiety, which is unusual for members of this class.

The absolute configuration of **4** is presumed here to be as shown on the basis of the likely incorporation of the more common L-glutamic acid unit. Attempts to determine the absolute configuration using the X-ray crystallographic data employing the Flack parameter *R*-value supported the proposed absolute configuration, but twinning observed in the crystal led to a relatively high *R*-value, resulting in an inconclusive independent assignment of the absolute configuration. Additional efforts were undertaken to confirm the absolute configuration of **4** by analysis of the ECD spectrum, as some members of this class have been examined and stereochemically assigned using such data (Singh et al. 1998; Hellwig et al. 2002). However, unlike these other compounds, the decalin moiety in this case has a *cis*-ring fusion, and it also does not contain a strong chromophore. As a result, comparison of the ECD spectrum of **4** to literature values for other members of the class (Hellwig et al. 2002) did not afford a conclusive assignment. Given that the tetramic acid moiety contains the only significant chromophore, it was felt that the CD spectrum would likely be dominated to some degree by that component. Thus, by analogy to an approach taken during studies of similar class members, the data were compared with those of a known compound, fuligorubin (Ley et al. 1992), and two reduced analogues (Hellwig et al. 2002), which contain a similar tetramic acid subunit derived from glutamic acid, and an achiral side chain in place of the decalin unit, but the results were again inconclusive. For example, the latter contain peaks of identical sign at 285–296 nm and 241–242 nm, while the spectrum of **4** shows a negative bisignate Cotton effect in this region [287 (+2.5) and 260 nm (−4.9)]. This difference is presumably due to the presence of the additional, complex, chiral decalin unit present in **4** that is not present in these analogues. Thus, no definitive conclusions were drawn regarding the absolute configuration on the basis of these data. The absolute configuration shown is judged to be most likely on the basis of the X-ray data and biogenetic considerations.

Metabolites of this class are derived from a mixed polyketide–amino acid origin and are known to exhibit diverse biological activities. For example, equisetin displays strong antibiotic activity against certain Gram-positive bacteria as well as phytotoxic effects and also inhibits HIV-1 integrase (Singh et al. 1998). Gabusectin was reported in the original patent to be active against *S. aureus* (Schiell et al. 2003).

Both of the organisms discussed here were initially assigned as members of the same genus on the basis of micromorphology, but subsequent partial sequence analysis of the ITS and 28S rDNA domains and BLAST queries of the GenBank database provided clear support for this taxonomic assignment only for one of these isolates (NRRL 44611; the isolate that produced compound **4**). Sequence analysis results for the other isolate do not match well with *Phialemonium*/*Phialemoniopsis* sequences in the database, matching most closely, but not convincingly, with sequence information for an unidentified member of the Bionectriaceae, so its identification is viewed as uncertain. The major secondary metabolites encountered from the two isolates were also quite different.

## Conclusion

These results show for the first time that a member of the genus *Phialemonium* (now *Phialemoniopsis*) produces bioactive secondary metabolites. Such compounds may be associated with phenomena observed in the past for *Phialemonium* spp., such as antagonistic effects against other fungi. Metabolites encountered in the current work were identified by NMR analysis, and structures of two of the compounds were independently verified by X-ray crystallographic analysis. While some uncertainty remains regarding the identity of one of the isolates, both of the source fungi are members of a large collection of isolates obtained mainly as colonists of fungal stromata or polypores collected in forests on the island of Hawaii. Chemical studies of these fungicolous isolates were undertaken as part of a long-term project to explore such fungi as distinctive sources of new biologically active natural products (Gloer 2007; Neff et al. 2012). The results presented here provide additional support for the potential of fungi from such sources in this regard.
